# Frugal innovation: a sustainable approach to simulated clinical skills training

**DOI:** 10.1038/s41415-026-9539-4

**Published:** 2026-04-24

**Authors:** Clement Seeballuck

**Affiliations:** Dundee School of Dentistry, University of Dundee, Dundee, United Kingdom

## Abstract

**Introduction** Environmentally sustainable practice must be considered when developing training interventions. This is particularly important with skills simulation – a field that, traditionally has significant consumables. This article presents a replicable case study of a clinical skills course with minimal cost and plastic waste.

**Background** A ‘rotary instrument skills' course was developed as part of the Dundee School of Dentistry revised curriculum, focusing on instrument ergonomics through tasks involving shape cutting, distinct from specific clinical scenarios, instilling transferable skills rather than replicating.

**Development** Core factors considered included safety, environmental sustainability, cost and pedagogy. Progressive cutting exercises were designed reusing typodont teeth from different courses. These were mounted in a novel jig created from popular building blocks allowing for pressure feedback and multiple reuses of each tooth.

**Impact** The course has run continuously since 2022. Students have responded positively to the virtually limitless cutting practice. Each jig initially cost less than £3, proving cost effective. Total plastic expenditure is minimal.

**Discussion and conclusion** This study demonstrates environmental sustainability in simulation. Alternatives like haptic simulators have no plastic expenditure after initial production, however, require significant infrastructure and cost. Frugal innovations provide potentially more equitable solutions for institutions with limited finances.

## Key points

- Environmental sustainability and cost-benefit analysis should be considered when planning new educational interventions.
- Frugal innovations have potential to enhance and supplement current teaching practices without a need for significant investment.
- Teaching innovations need to consider modern pedagogy when designing training exercises.

## Introduction

Teaching practice cannot be considered static. There is an ever-present drive to improve and adapt. Blending modern pedagogy with technological innovation presents new opportunities to revolutionise how we teach – the impact of haptic simulators is a testament to this.^1^^,^^2^ There are now more ways than ever before to train the next generation of clinicians, and this certainly extends to clinical skills.

However, when considering innovation, there are key factors ubiquitous globally that need to be addressed: cost and environmental sustainability. These are important drivers that underpin how we should innovate teaching. Cost is also important for the mitigation of educational inequality. Innovations and new methods of teaching should allow optimal teaching to be just as achievable for institutions with the most limited resources. It is a lamentable reality as well that resources and funding available for universities are finite, posing a significant challenge for dental education.^3^ Environmental sustainability can be particularly challenging for simulated skills teaching as activities here tend to require significant plastic consumables for tooth preparation practice. Revision of simulated skills there has significant potential to reduce plastic waste.

This article details a novel clinical skills course, using ‘frugal innovation' to train students on the appropriate use of rotary instruments such as handpieces. This course considers environmental sustainability and has a dramatically smaller carbon footprint when compared to similar cavity cutting exercises, due to the extensive reuse of typodont teeth. The course is easily replicable across universities.

### Background

In 2019, the Dundee School of Dentistry implemented a new ‘4D' (knowledge, skills, character, and meta-learning) curriculum.^4^ This created an opportunity to radically rethink training practice and embrace modern pedagogy. A component of this included the development of a ‘rotary instrument skills course' to be integrated into the first year of the degree. This course aimed to teach ergonomics handpiece use through tasks involving shape cutting distinct from any specific clinical scenario: instilling transferable skills rather than replicating. The course was planned to take place before the traditional operative carious lesion management practical classes, with the intent of fostering ‘good habits' such as posture and safe practice, together with coordination and dexterity when manipulating instruments in the oral cavity. Therefore, the overall purpose was to support rather than replace existing teaching.

## Development

### Equipment

Four key domains were considered when developing the course tools:

- Staff and student safety
- Minimising cost
- Aligning to sound educational theory
- Environment sustainability and mitigation of carbon footprint.

There is currently a considerable focus on environment sustainability, and this extends to simulated skills within dentistry and beyond. Mitchell *et al.,*^5^ although more medically focused, have developed a toolkit to support environmentally sustainable course design and Marsack *et al.* have recently published a systematic review aptly summarising the current environment as well as discussing the ‘five Rs framework' of environment sustainability (reduce, reuse, recycle, research, and rethink).^6^ When considering these aspects, it was agreed that the course should have little to no plastic expenditure following initial setup.

For part of the course, a series of exercises was designed, involving cutting shapes repeatedly, progressing to more complex shapes when individuals feel they are ready. The ideal scenario for this was to allow students the freedom to explore and learn without any constraints related to materials shortages. Several materials were considered for cutting shapes, including bespoke blocks, 3-dimensional printed exercises, expired denture acrylic, and trays. The majority of these are not suitable however due to safety issues or effort to produce. All materials that did not have readily available material safety data sheets explicitly stating it was it was safe to grind and generate particulates were immediately rejected.

**Fig. 1:**
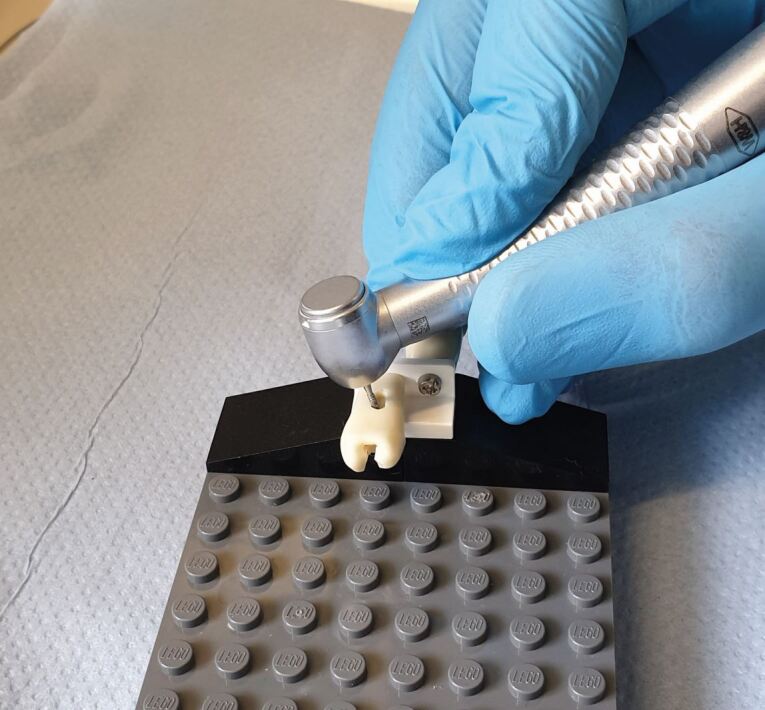
The tabletop jig configuration demonstrating finger rest and handpiece ergonomics and control

It was eventually decided to use typodont teeth that had already been used in different courses. These were mounted perpendicularly in a novel jig created from popular building brick toys allowing for the cavity preparation to be carried out on the previously unused root surfaces (Fig. 1). This configuration allowed the students to prepare multiple surfaces by turning the tooth.

**Fig. 2:**
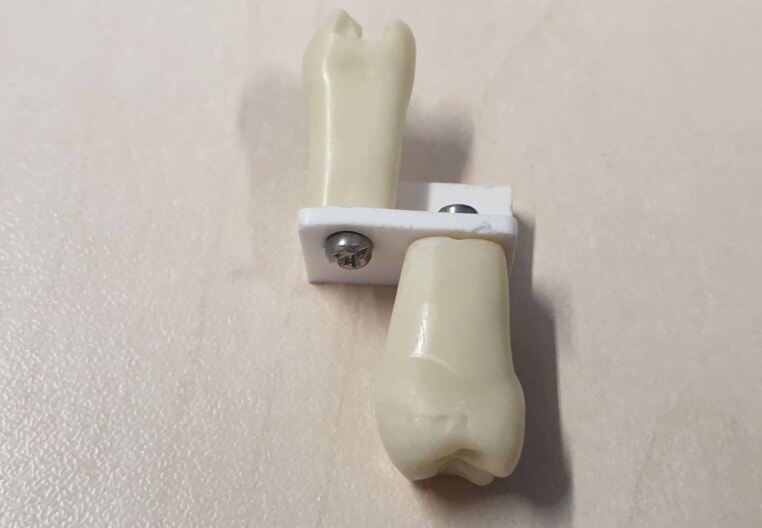
A small brick is used for connecting the typodonts to the baseplate. Students receive feedback due to the tendency of the brick to disconnect under excessive force

There were several advantages to using the building bricks:

- Cost – the individual bricks can be purchased online in large quantities. Due to the high volume produced, the total cost of the bricks was less than £3 per jig
- Availability – the market leading building blocks are effectively ubiquitous internationally. This means that similar setups can be employed with relative ease in most institutions
- Modularity – due to the endless possible building configurations, the jig could be assembled to closely replicate anterior or posterior tooth positions. Brick configurations can also be used to increase complexity as the students' progress
- Durability – by procuring bricks from the market leading brand, there was an inherent manufacturing precision. The bricks can be used repeatedly without failure or breakage
- Environment sustainability – the market leading brand are committed to greener practice and aim to have net zero carbon emissions by 2050
- Pressure feedback – this unexpected advantage became apparent during use. As the typodonts were mounted onto a small brick (Fig. 2), excessive pressure caused the bricks to fall apart. The clinicians involved in this practice likened the maximum pressure needed to cause disassembly to be similar to the maximum pressure you would want to exert when using a handpiece clinically.

**Fig. 3:**
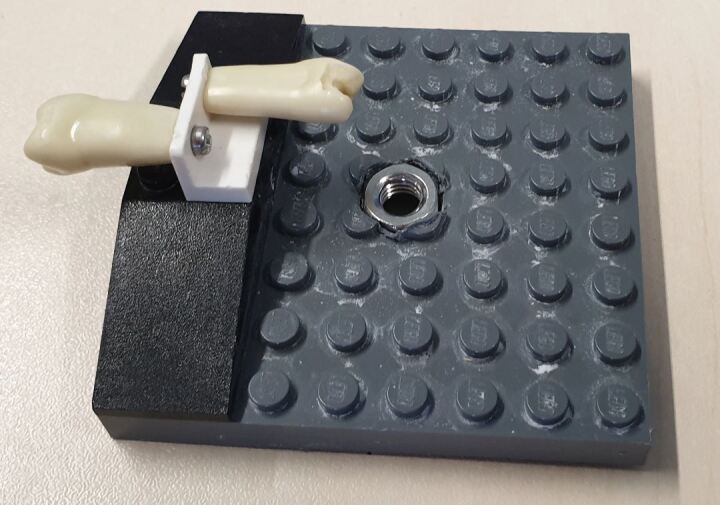
The current iteration of the mounting jig used for training. The baseplate size, with the addition of the nut allows for use within a phantom head

A final revision was made to the apparatus by incorporating an M6 nut to the central baseplate (Fig. 3). This nut is readily available in any hardware store and allows the baseplate to be screwed to the majority of phantom heads available. This current iteration now means that the course can run exercises both inside a phantom head and on a tabletop with considerable potential for customisation due to the inherent modularity of construction bricks.

### The exercises

When developing the classes, the ‘findability, accessibility, interoperability, and reusability of data principles' were considered: teaching should have feedback, be active, tailored to the individual and be relevant to their course.^7^ The exercises follow a ‘progression' format rather than a specific class itinerary. Students are allowed to develop at their own pace, moving onto the next exercise when they feel ready. Each Progression increases complexity:

- Progression 1 – direct vision, simple shapes
- Progression 2 – direct vision, complex shapes
- Progression 3 – indirect vision, simple shapes
- Progression 4 – indirect vision, complex shapes.

**Fig. 4:**
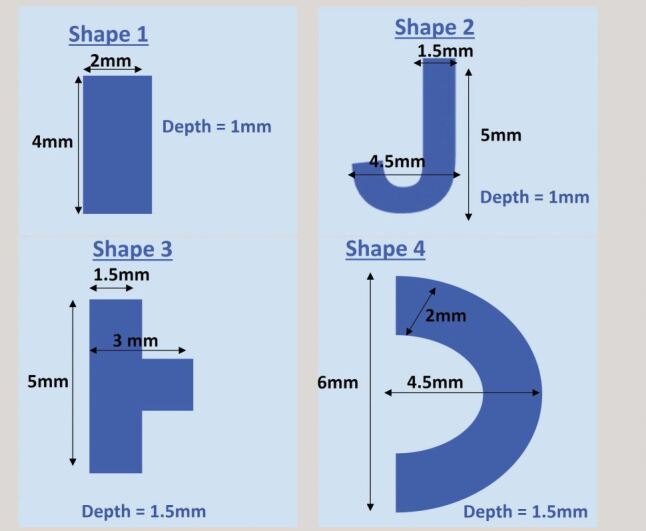
Examples of simple shapes

**Fig. 5:**
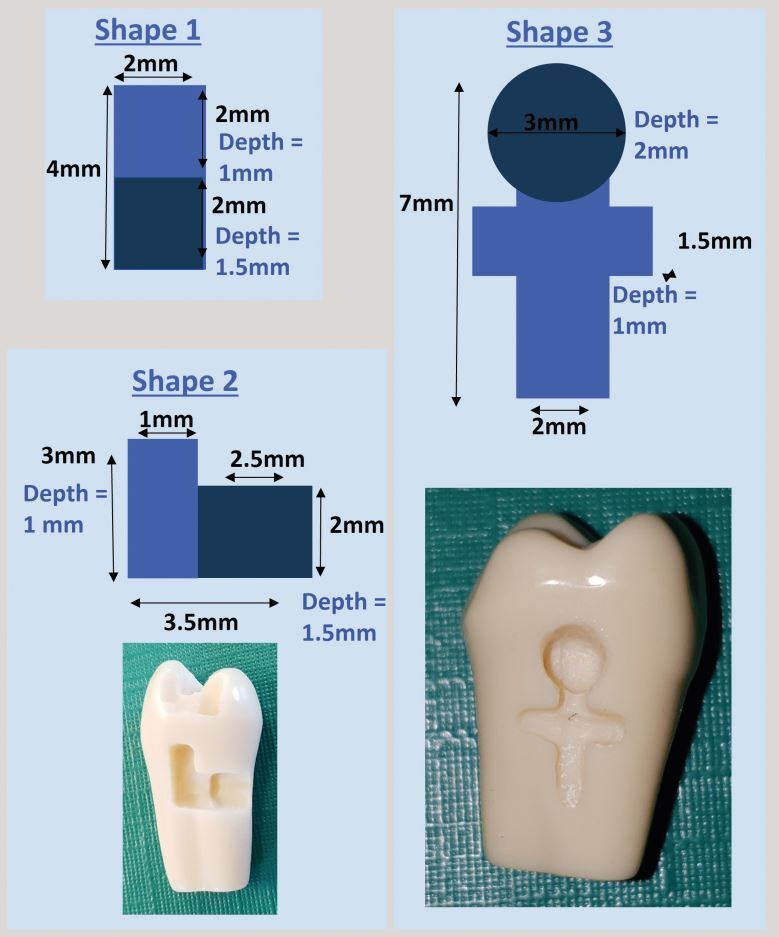
Examples of more complex shapes

Asymmetric shapes, purposefully distinct from carious lesions were developed for the exercises. The rationale for this is that the course focuses on technical skill and is a precursor to all cutting classes. The shapes use specific measurements (Fig. 4, Fig. 5), which the students measure precisely using callipers. This allows students to ‘calibrate' their actions to the end result. Once students become proficient at estimating dimensions, the callipers are removed, and students begin to estimate with the more traditional ‘bur widths'.

Although the course employs several technically challenging shapes, the final assessment relates to precise cutting of one simple shape with direct and indirect vision.

Students are also supported throughout the exercises via peer-assisted learning.^8^^,^^9^ Final-year students are trained in how to give feedback through a workshop and then support the first-year students with advice relating to instrument ergonomics and posture. This allows the first-year students to get significantly more feedback and provides the final years with insight into working in a supervisory capacity. However, all final assessment of performance is undertaken by the supervisory staff.

### Impact

The course has been running continuously since 2022, and students have responded positively to the virtually limitless opportunity to practice cutting. Students who require extra practice can also use as many teeth as they want in extra practice sessions. This provides an environment unbound by material limitations that would otherwise be the case with purchasing new typodonts.

The building blocks have held up well for several years without needing to be replaced. With each jig having an initial cost of less than £3, this has proved very cost effective. Total plastic expenditure is also minimal. Two teeth are procured for each student for their end of course assessment, ensuring standardised exam conditions. All other teeth used throughout the course are reused. On average, students prepare four cavities per session. This equates to a saving of 16 typodonts per student annually, saving significant plastic and cost.

In light of the environmentally sustainable practice employed, the course was awarded the Henry Schein ‘Oral Health Professional Educators' Practice Green Award' in the category of Faculty Procurement and Product at the the Association for Dental Education in Europe annual conference in 2023.

## Discussion and conclusion

This case study demonstrates a way of innovating clinical training without significant expenditure or plastic waste. This intervention has been reviewed and implemented in the curriculum higher education institution and has continued for a number of years with minor modifications.

There are of course alternative methods of training such as haptic simulators that have no plastic expenditure after initial production. Haptic simulators have a growing evidence base confirming their effectiveness as training tools, but also require significant initial expenditure, maintenance packages, and a suitable infrastructure.^1^^,^^10^^,^^11^ The training tools discussed in this article do not have any of these issues. It must however be acknowledged that haptic simulators and physical cutting training tools aim to achieve similar training in very different ways. Therefore, the author considers these training tools as complementary when both are available. The inherent modularity of the bricks means that different configurations can be employed that increase or decrease the amount of pressure required. This in effect provides a basic haptic element to the training and also can allow for increasing difficulty of exercises as the student progresses. The versatility of the bricks also means that specific scenarios can be replicated that would not be possible with a traditional typodont configuration.

An argument can be made that these exercises do not resemble tooth morphology or specific clinical exercises. However, they do replicate skills such as precise preparation and indirect vision. It also must be noted that most typodont and phantom head setups do not have tongues, movement, saliva or materials that exactly replicate oral tissues. A very important question therefore is ‘how realistic does a training exercise need to be in order to be effective for developing transferable clinical skills?'. This is a crucial question when considering simulation and further robust investigation is warranted. To date, the course has received very positive feedback from students and staff involved; however, this is more anecdotal. It will be important to formally evaluate this intervention considering improved outcomes, student perceived benefit and how it well the skills translate to progressive clinical skills courses.

Frugal innovation solutions by their very nature are easy to replicate and accessible for most institutions as they require little expenditure. This is particularly important when considering equitable education. If effort is only expended on developing educational resources which require significant funding, then there is a very real danger of creating an educational divide. Ultimately everyone should be entitled to the best education possible. Therefore, it is crucial that low-cost interventions are shared, and their effectiveness robustly evaluated

## Data Availability

As this article details a novel method of teaching practice and does not relate to specific research, there are no links to additional data.
